# Investigating the Influence of Varied Light-Emitting Diode (LED) Wavelengths on Phototactic Behavior and Opsin Genes in Vespinae

**DOI:** 10.3390/ani14111543

**Published:** 2024-05-23

**Authors:** Xiaojuan Huang, Tong Zhou, Hasin Ullah, Danyang Zhu, Yan Tang, Hongli Xu, Hang Wang, Jiangli Tan

**Affiliations:** Shaanxi Key Laboratory for Animal Conservation, Key Laboratory of Resource Biology and Biotechnology in Western China, Ministry of Education, College of Life Sciences, Northwest University, Xi’an 710069, China; huangxiaojuan@stumail.nwu.edu.cn (X.H.); zhoutong@stumail.nwu.edu.cn (T.Z.); hasenullah888@yahoo.com (H.U.); zhudanyang@stumail.nwu.edu.cn (D.Z.); tangyan1@stumail.nwu.edu.cn (Y.T.); 202233084@stumail.nwu.edu.cn (H.X.); wanghang@stumail.nwu.edu.cn (H.W.)

**Keywords:** *Vespula germanica*, *Vespa analis*, *Vespa basalis*, phototaxis, opsin genes

## Abstract

**Simple Summary:**

This research aims to explore the phototactic behavior and key opsin genes associated with Vespinae. The results showed that the two species, *Vespula germanica* and *Vespa analis*, exhibited varying photophilic rates under different wavelengths of light, suggesting that light wavelength significantly affects their phototactic behavior. Additionally, the opsin genes of the most aggressive hornet, *Vespa basalis*, have been sequenced. There are only two opsin genes, one for UV light and the other for blue light, and *Vespa basalis* lacks long-wavelength visual proteins. However, they exhibit peak phototaxis for long-wavelength light and instead have the lowest phototropism for UV light. This suggests that the visual protein genes have a complex regulatory mechanism for phototactic behavior in Vespinae. Our findings provide a sound theoretical basis for further investigation of visual expression patterns and phototactic mechanisms in Vespinae.

**Abstract:**

The phototactic behavior of insects is commonly used to manage pest populations in practical production. However, this elusive behavior is not yet fully understood. Investigating whether the opsin genes play a crucial role in phototaxis is an intriguing topic. Vespinae (Hymenoptera: Vespidae) are a common group of social wasps that are closely associated with human activities. Efficiently controlling wasp populations while maintaining ecological balance is a pressing global challenge that still has to be resolved. This research aims to explore the phototactic behavior and key opsin genes associated with Vespinae. We found significant differences in the photophilic rates of *Vespula germanica* and *Vespa analis* under 14 different light conditions, indicating that their phototactic behavior is rhythmic. The results also showed that the two species exhibited varying photophilic rates under different wavelengths of light, suggesting that light wavelength significantly affects their phototactic behavior. Additionally, the opsin genes of the most aggressive hornet, *Vespa basalis*, have been sequenced. There are only two opsin genes, one for UV light and the other for blue light, and *Vespa basalis* lacks long-wavelength visual proteins. However, they exhibit peak phototaxis for long-wavelength light and instead have the lowest phototaxis for UV light. This suggests that the visual protein genes have a complex regulatory mechanism for phototactic behavior in Vespinae. Additionally, visual protein sequences have a high degree of homology among Hymenoptera. Despite the hypotheses put forward by some scholars regarding phototaxis, a clear and complete explanation of insect phototaxis is still lacking to date. Our findings provide a strong theoretical basis for further investigation of visual expression patterns and phototactic mechanisms in Vespinae.

## 1. Introduction

Phototaxis, the phenomenon wherein insects respond to light stimuli by moving towards (positive phototaxis) or away from (negative phototaxis) a source of light, is a behavior that has evolved as an inherent characteristic in insects [1]. This behavior is functionally adaptive as it regulates light exposure and aids in spatial orientation [2]. It influences various aspects of insect life, including mating, feeding, life cycle, and reproductive modes [1,2,3]. The response of insects to light is influenced by the wavelength of light [4]. Extensive research in recent years has explored the phototactic behavior of different insect species, with a particular focus on pests [5,6]. Hymenoptera, a group that includes bees and wasps, exhibits sensitivity peaks in the visual spectrum at 340 nm, 430 nm, and 535 nm, indicating a predominantly trichromatic visual system [7,8]. For instance, the honeybee (*Apis mellifera*) is sensitive to light at 344 nm, 436 nm, and 556 nm [9]. Phototactic behavior is also influenced by light intensity and is often used to assess an animal’s ability to discriminate between different levels of light intensity [10,11]. The underlying mechanisms of phototaxis are not hardwired and can be modulated by various external factors, such as light, wavelength, intensity, sex, developmental stages, environmental conditions, and biological rhythms [12,13,14,15,16,17]. Insect phototactic behavior is also subject to modulation during biological rhythms, with different species exhibiting peak light-up periods at specific times [18,19]. Scholars have proposed several hypotheses to explain insect phototaxis, such as light orientation, biological antennae, light interference, and light stress hypothesis [20,21,22,23]. This research aims to further investigate the intricate nature of insect phototaxis, shedding light on its underlying mechanisms and contributing to our understanding of this behavior.

Vision is an important physical sense in insects, facilitating foraging, mating, and defense against predators [24]. The ability of insects to recognize different spectra and produce corresponding visual responses depends critically on the visual pigments present in the photoreceptors inside the compound eye. These pigments consisted of photosensitive chromophores and optic proteins [25]. In most insects, there is only one form of photosensitive chromophore, and its structure is relatively simple. Therefore, the diversity of spectral recognition in insects relies solely on the sequence of optic proteins. Opsin, a G-protein-coupled receptor characterized by the presence of seven transmembrane domain structures, is the primary visual protein [26]. Visual proteins can be classified into seven subfamilies based on their specific functions and molecular structures: neuropsin, encephalopsin, retinal photoisomerase, Go-coupled retinal proteins, Gq-coupled retinal proteins, and vertebrate visual and non-visual retinal proteins [26]. Optic proteins can be further classified into non-visual opsins and visual opsins based on their roles in visual imaging. Opsins can be categorized as rhodopsin or visinin based on their molecular properties, which differ in the amino acid sequence at residues 122 and 189 [27]. Previous studies have shown that insect opsins can be divided into three clades based on the spectral peaks of the pigments: long-wavelength-sensitive opsin (LW opsin, peak absorbance 500–600 nm), blue-sensitive opsin (blue opsin, peak absorbance 400–500 nm), and ultraviolet-sensitive opsin (UV opsin, peak absorbance 300–400 nm) [28]. Insects often adjust their spectral sensitivity range to match the spectrum of their environment, leading to duplication, loss, and variation in the opsin genes. For example, certain insects in the Coleoptera order have lost optic proteins that are sensitive to short-wavelength light [29]. Variations, losses, and duplications of optic protein genes have also been observed in species such as Drosophila, mosquitoes, and butterflies [30]. Research on the function of the opsin genes has become important. UV opsin is important for flower recognition by honeybees [31] and is involved in the migratory orientation of the monarch butterfly *Danaus plexippus* [32]. In experiments with psyllid *Diaphorina citri*, reducing the expression of three opsin genes resulted in reduced phototactic efficiency to UV, green, and blue lights [33].

The subfamily Vespinae, belonging to the family Vespidae of Hymenoptera, are social insects present worldwide and attack flies, moths, locusts, cicadas, and beetles. Vespinae are often regarded as pests due to the threats they pose to human production, health, and life, such as attacking humans, preying on bees, destroying fruits, and contaminating food [34]. Currently, pest management has traditionally relied on integrated pest management, which utilizes sex pheromones and phototactic behavior, which is a sustainable approach in production practice [35,36], While research has yielded results on the phototactic behavior of insects and pest control strategies based on it, the phototactic behavior of wasps in the subfamily Vespinae has been understudied compared to other taxa.

In this study, we aimed to investigate the phototactic behavior of two Vespinae species, *Vespula germanica* (nest underground) and *Vespa analis* (nest on the tree). We analyzed and compared their phototactic behavior at six different times of the day using a range of LED lights with distinct colors or wavelengths. Our main objective was to identify patterns and gain insights into the phototactic behavior of Vespinae. Additionally, we fully sequenced and analyzed the UV and blue light opsin genes of *V. basalis* (the most aggressive species of hornets). Using bioinformatics techniques, we sequenced the deduced amino acid sequences of the opsin genes. Combining with the hymenopteran data online (NCBI) (Table S9), a phylogenetic tree was constructed, which revealed both the conservation of the opsin genes and the phylogenetic relationships among the species.

## 2. Materials and Methods

### 2.1. Insect Maintenance and Experimental Conditions

*Vespula germanica* and *Vespa analis* were respectively collected from Maowusu Desert, Shenmu City, Yulin, Shaanxi, China, and Duhui Village, Chang’an District, Xi’an, Shaanxi. The collection was conducted in August and September 2021. Wasps were reared in a clear acrylic box at an ambient temperature of 25 ± 2 °C, a relative humidity (RH) of 60–70%, and a 12:12 light-dark photoperiod (light cycle: 6:00–18:00; dark cycle: 18:00–6:00). The insects were fed with honey solution and apples.

### 2.2. Monitoring Phototactic Behavior

**Figure 1:**
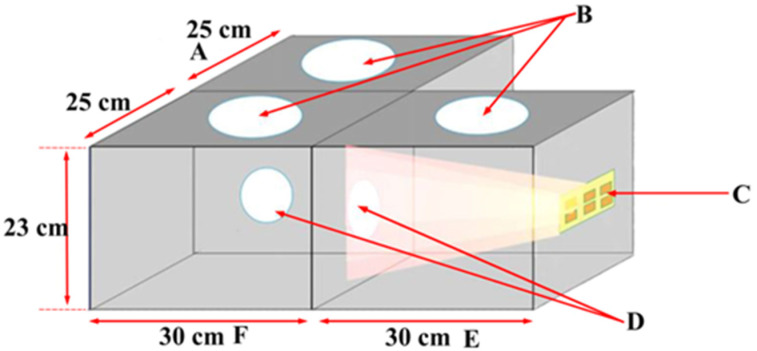
Apparatus for monitoring the phototactic response of *Vespula germanica* and *Vespa analis*: (A) light-sheltered reaction chamber (dark area); (B) picking up and releasing insects around the mouth; (C) LED light; (D) pathway for light or/and wasps; (E) phototactic chambers (light zones with internally mounted light sources); and (F) waiting room.

The phototaxis monitoring system (Figure 1) was designed based on Huang et al. (2023) and Jiang et al. (2023) [37,38]. It included a phototactic reaction chamber (light zone), a static chamber, and a dark chamber (dark zone). The system was made of transparent Plexiglas and measures 30 cm × 23 cm × 25 cm. The phototactic chamber and the light-avoidance chamber were positioned at 90° to ensure minimal interference between both. A circular pathway with a diameter of approximately 6 cm was provided between the static and phototactic chambers and the light-avoidance chambers. Above each chamber was a circular opening, approximately 10 cm in diameter, for the release and retrieval of insects. The bottom and top walls were covered with opaque cardboard to minimize exposure to light from the other chambers. 

A white LED light of the light source with a spectral wavelength ranging from 360 to 635 nm (Xuzhou Aijia Electronic Technology Co., Ltd. (Jiangsu, China)) was placed along one side of the glass with a 5 W 12 V lithium-ion battery LED light board. We customized 14 different models with wavelengths ranging from 360 to 365 nm (UV) and 380 to 385 nm (ultraviolet light), 400 to 410 nm (violet light), 420 to 430 nm (blue-violet light), 440 to 445 nm (dark blue light), 460 to 475 nm (blue light), 490 to 505 nm (faint green light), 515 to 525 nm (bright green light), 530 to 545 nm (green light), 550 to 565 nm (green-yellow light), 570 to 590 nm (yellow light), 600 to 610 nm (orange-red light), 625 to 635 nm (red light), and 400 to 840 nm (composite white light that mimics sunlight). During the operation of the LEDs, the temperature varied by ±1 °C. The LED lights were tested with a Polaroid polarizer to confirm that LEDs do not produce polarized light. To measure illumination intensity and spectrum, we used an Avos V3 photometer to more precisely measure the light intensity inside the monitoring cages.

### 2.3. Behavioral Response of Vespula germanica and Vespa analis to Different Spectral Wavelength Light

To explore the phototactic behavior characteristics of wasps, we conducted phototropic experiments on male and female *Vespula germanica* and *Vespa analis*. The period from 7:00 to 19:00 was selected based on their overall activity pattern. Each experiment lasted for 2 h, comprising a 1 h dark reaction phase, a 45 min light reaction phase (with a light intensity of 160 lx), and a 15 min subsequent treatment phase [39]. At 7:00, 30 female wasps were placed in a static chamber. The circular pathway connecting the phototactic and light-avoidance chambers was then blocked, and the entire experimental setup was covered with a black cloth to conduct the dark reaction for 1 h. Following the dark reaction, the light source and circular pathway were activated, and the experimental setup remained covered with black cloth to prevent interference. The light reaction was then carried out for 45 min. Then, the number of insects in the phototactic and light-avoidance chambers was counted for 15 min. Finally, the chambers were wiped with alcohol pads and cleaned. Following this, we placed 30 test insects into the resting chamber for the next experiment. This process was repeated until the experiment concluded at 19:00. Each experimental setup could conduct six sets of experiments per day. The light reaction periods were from 8:00 to 8:45, 10:00 to 10:45, 12:00 to 12:45, 14:00 to 14:45, 16:00 to 16:45, 16:00 to 16:45, and 18:00 to 18:45, with each light type being treated for four consecutive days. A total of 24 replications were conducted. Phototactic behavioral response rate (%) = (number of wasps in the reaction chamber/total number of wasps) × 100. All experiments were repeated thrice; photophobic rate percentage (%) = (number of light-avoidance reaction chamber wasps/total number of wasps) × 100; and percentage of active wasps (%) = (number of wasps in phototactic and light-avoidance chambers/total number of wasps) × 100.

### 2.4. Cloning of Opsin Genes

The most aggressive hornet, *Vespa basalis*, being collected from Qianyang (Baoji, Shaanxi), was selected for cloning its opsin genes. Their compound eyes, being dissected soon, were frozen in liquid nitrogen for the following experimental steps. Total RNA was isolated using a Trizol extraction kit No. B511321 (Sangon Biotech, Shanghai, China), following the manufacturer’s protocol. A NanoDrop 2000 spectrophotometer (Hitachi, Tarrytown, New York, NY, USA) was used to perform spectroscopic quantification. Primers were designed based on the cDNA sequences (Table 1). The following reagents were added to 0.2 mL PCR tubes: 5 μL RNA sample, 1 μL random primer, 1 μL ddH_2_O, 70 °C warm bath for 5 min. Ice bath for 2 min; centrifugation: 2.0 μL 5X First-Strand Buffer, 0.5 μL 10 mmol dNTP, 0.25 μL RNase inhibitor, 0.25 μL Reverse Transcriptase, 10.0 μL total volume, 42 °C warm bath for 60 min, and 72 °C warm bath for 10 min. Then, the PCA reaction was performed. The results were observed through 1% agarose gel electrophoresis. The PCR products were recovered and sent to Sangon Biotech (Shanghai) Co., Ltd. for the complete sequencing of the DNA.

### 2.5. Statistical Analysis, Sequence Alignment, and Phylogenetic Tree

The statistical analyses were performed using SPSS v20 [40]. Tukey’s HSD was used for multiple comparison tests. In every test, significance levels were denoted by * (0.01 < *p* < 0.05) and ** (*p* < 0.01). The molecular weight and isoelectric points, functional sites and domains, Transmembrane topology and structure prediction, and second-level structure of two opsin proteins from *Vespa basalis* were predicted respectively using ExPASy’s [41] ProtParam tool (https://www.expasy.org/resources/protparam, accessed on 10 September 2022), (ScanProsit (https://www.expasy.org/resources/scanprosite, accessed on 5 September 2022) and NCBI (https://www.ncbi.nlm.nih.gov/cdd, accessed on 7 September 2022), DeepTMHMM v1.0.20 (https://dtu.biolib.com/DeepTMHMM, accessed on 12 September 2022) [42], and SOPMA (https://npsa-pbil.ibcp.fr/cgibin/npsa_automat.pl?page=npsa_sopma.html, accessed on 15 September 2022). For phylogenetic analyses, amino acid sequences of long- and short-wavelength opsin genes, each with twenty-two insect species mainly belonging to the order Hymenoptera, were obtained from the NCBI (Table S9) database and compared with our study data of *Vespa basalis* using blast analysis. The 3D structure of opsin genes was predicted by the online analysis tool SWISS-MODEL (https://swissmodel.expasy.org/, accessed on 18 September 2022) [43]. To determine the opsin amino acid sequence of the homologous species with high identity, the online analysis tool T-Coffee (https://tcoffee.crg.eu/, accessed on 20 September 2022) was used to perform a consistency analysis of the opsin amino acid sequence [44]. The MEGA v11 software [45] was used to construct a phylogenetic tree by maximum likelihood with 1000 replicates.

## 3. Results

### 3.1. Phototactic Rhythmicity of Vespula germanica at Different Times

**Figure 2:**
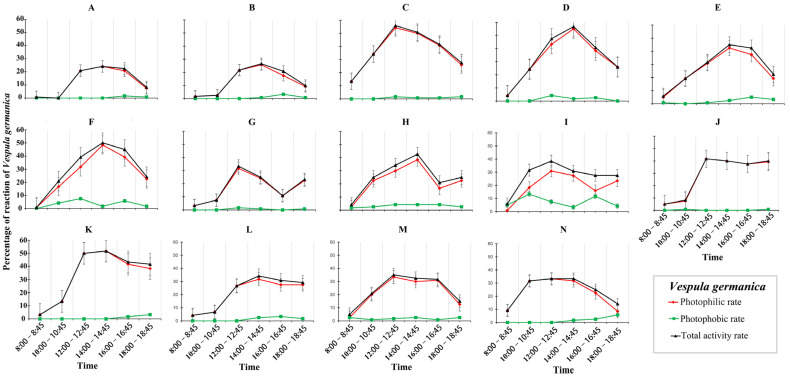
The photophilic, photophobic, and total activity rate of *V. germanica* at different time intervals under monochromatic light: (**A**) 360−365 nm; (**B**) 380–385 nm; (**C**) 400−410 nm; (**D**) 420−430 nm; (**E**) 440−445 nm; (**F**) 460−475 nm; (**G**) 490−505 nm; (**H**) 515−525 nm; (**I**) 530−545 nm; (**J**) 550−565 nm; (**K**) 570−590 nm; (**L**) 600−610 nm; (**M**) 625−635 nm; (**N**) 400−840 nm.

The phototactic rates of *Vespula germanica* varied significantly across the six time periods for each light, indicating a rhythmic behavior. *V. germanica* exhibited varying phototactic rates across different wavelengths of light, with active periods of different lengths. The lowest photophilic rates were mainly concentrated in the morning, while the highest photophilic rates appeared entirely in the noon and the late afternoon. The lowest and highest photophilic rates appeared at the same or similar periods under different wavelengths, but the specific phototactic trends and rates were quite different. *V. germanica* exhibited varying phototactic strengths for different wavelengths of light, as evidenced by the considerable variation in specific phototactic trends and rates, despite the lowest and highest rates occurring at similar times. Additionally, the phototactic trends under neighboring wavelengths of light were similar (Figure 2 and Table S1).

The photophilic and photophobic rates were not significantly different most of the time under both UV lights, with significant differences only at mid-day, late afternoon, or afternoon hours. Under violet, blue-violet, blue, and yellow light, photophilic and photophilic rates were significantly different except in the morning. Significant differences in photophilic and photophobic rates over the whole spectral range of green light (490–565 nm) were mainly observed at mid-day, late afternoon, and evening, which is a unique photophilic rhythm in the face of green light, i.e., photophilic shifted from a downward trend to an upward trend in the afternoon, suggesting that a second peak of photophilic may be formed in the evening and onwards. The differences in photophilic and photophobic rates within the two red light types and the composite light types were significant, except for morning and evening, suggesting that the phototactic activity of *V. germanica* to long-wavelength light may only be active in the morning to afternoon hours. Overall, photophilic and photophobic rates had the highest and most significant differences among the 14 light types at mid-day and late afternoon, whereas there were mostly no significant differences in the morning and evening (Figure 2 and Table S1).

By analyzing the photophobic rate of the different periods, we found that *V. germanica* exhibited low photophobic rates at different times of the day, with a generally smooth trend. It was only significantly different in the UV light (380–385 nm), green light, and composite light (Table S2). Although there were significant differences in the photophobic rates of the three light types, most of them remained at a low level. Therefore, it is reasonable to assume that *V. germanica* does not exhibit a significant photophobic rate (Figure 2).

The analysis of the total activity rate of *V. germanica* at different times all day showed significant differences in the active rates (Figure 2 and Table S3). The analyses indicated that the activity rate and change trend were similar to that of the photophilic rate. This was because the photophobic rate is generally low or even zero, and the trend of change was mostly smooth without significant fluctuations (Tables S4 and S6). As *V. germanica* did not exhibit photophobic behavior, its activity was determined by the photophobic rate (Table S5).

### 3.2. The Phototactic Behavior of Vespula germanica under Different Wavelengths of Light

**Figure 3:**
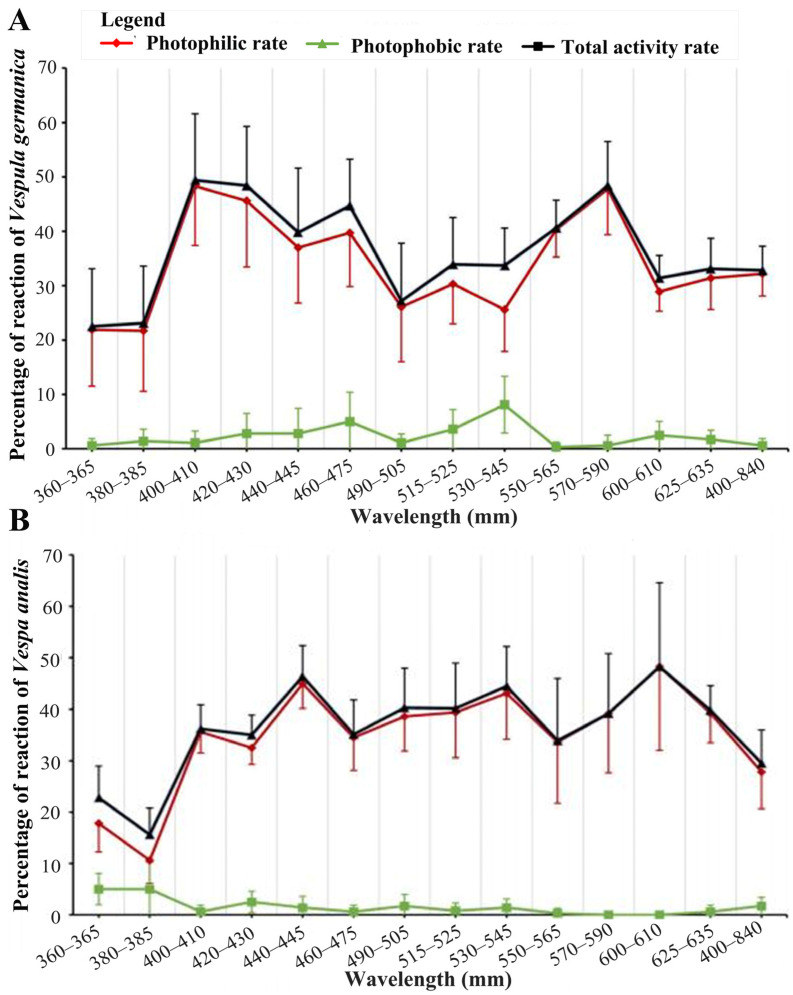
Spectral response curves of (**A**) *Vespula germanica* and (**B**) *Vespa analis*.

We classified 14 types of light based on their wavelengths. *Vespula germanica* had the lowest phototropism rate of about 20% when it came to sensitivity to UV light. Meanwhile, green, red, and white light have phototropism rates of about 30%, which were significantly higher than that of UV light. Blue light had the highest phototropism rate at 38.4%, indicating a stronger preference compared to the other types of light. In conclusion, the sensitivity and tendency of *V. germanica* to different wavelengths of light varied, and a bimodal response curve emerged, with violet light and yellow light being the most preferred, with an average phototactic rate of 47.0% and 44.1%, respectively (Figure 3 and Table 2).

*Vespula germanica* exhibited weak responses to 14 different light sources, with a generally low photophobic rate. The photophobic response was less pronounced compared to the photophilic response, and the curve exhibited a gentler slope. However, there was a significant variation in the photophobic rate among different wavelengths. The curve displayed multiple peaks, with three prominent peaks observed in blue (460–475 nm), green (530–545 nm), and orange-red (600–610 nm) light. It is important to note that the highest photophobic rate was observed under green light, while the photophilic rate was at its lowest (Figure 2). The difference in the photophobic rate can primarily be attributed to the considerably higher photophobic response to green light compared to other light wavelengths.

The total activity rate of *V. germanica* and the photophilic rate exhibited a high degree of consistency. The photophobic rate was only higher in the green light, which prevented *V. germanica’s* total activity rate curve from dipping as much as the photophilic rate curve. This led to the formation of a distinct result in the formation of a new trough (Figure 3).

*Vespula germanica* demonstrated notable variations in both photophilic and photophobic rates across all 14 light sources. The photophilic rate exhibited a much stronger response compared to the photophobic rate. Specifically, a significant difference was observed between the photophilic rate and the overall activity rate only under green light. The overall activity rate of *V. germanica* was primarily influenced by the photophilic rate, while the photophobic rate remained relatively weak across most light conditions. Notably, significant disparities were observed between the photophobic rate and the overall activity rate for all 14 light sources. These findings align with the results obtained from comparing the photophilic and photophobic rates (Figure 2 and Table 3).

### 3.3. Phototactic Rhythmicity of Vespa analis at Different Times

By studying the phototactic rhythm of *Vespa analis*, it was found that the phototactic rate of *V. analis* was significantly different in six time periods under each type of light, indicating that the phototactic behavior of *V. analis* had a rhythmic nature. *V. analis* had different phototactic rhythms under different wavelengths of light, the phototactic period varied from long to short, and the photophilic rate was always high from morning to evening under some lights, which indicated that the phototactic period of *V. analis* might include more periods. Then, the lowest photophilic rate occurred mostly in the morning, similar to *Vespula germanica*, but the highest photophilic rates were not concentrated in the mid-day and late afternoon as in *V. analis* but occurred in the morning and evening. The minimum and maximum photophilic rate of *V. analis* in different wavelengths of light occurred at similar times, but the specific phototactic tendency and photophilic rate varied considerably, reflecting that *V. analis* had different phototactic strengths for different lights (Figure S1 and Table S1).

Analysis of the total activity rate of *V. analis* in different periods showed significant differences in the six time periods. The trend was consistent with the phototactic rate, as the photophobic rate of *V. analis* was generally low or even zero. The phototactic rates of *V. analis* exhibited no significant difference across the six time periods of the 14 light types, except for the mid-day period under the composite light. Furthermore, when the significance of the difference between the photophobic rate and the total activity rate of *V. analis* was compared, we found that the photophobic rate and total activity rate of *V. analis* were significantly different in most of the periods. In conclusion, it is further demonstrated that the variation in the total activity rate of *V. analis* is mainly dependent on the photophilic rate (Figure S1).

The analysis of the photophobic rate of *V. analis* revealed that the photophobic response was much weaker than the phototactic response at different wavelengths, and in some cases, there was no photophobic response at all. The photophobic rate varied significantly across different wavelengths, and the photophobic rate curve had multiple peaks. There were six sensitive peaks located in the ultraviolet, blue-violet, microgreen, green, and composite light regions. The highest photophobic rate was observed in the ultraviolet regions. The significance of the difference in photophobic rate mainly stems from these two types of light. It is important to note that the photophobic rate was highest while the photophilic rate was lowest in UV light with a wavelength of 380–385 nm. The results indicated that the total activity rate of *V. analis* is mainly dependent on the photophilic rate, as evidenced by the consistent curves observed for both parameters across all 14 light species tested (Figure S1).

By analyzing the significance of the difference between the different periods for all monochromatic lights, it was found that the phototactic rate of *V. analis* was significantly different between the different periods for blue light, bright green light, and mixed light (Table S2). Comparative analyses of the photophobic rate of three light sources at different times of day showed that under blue light, it had the highest photophobic rate in the morning with a significant difference; under bright green light, it had the highest photophobic rate in the morning with a significant difference; and under compound light, it had the highest photophobic rate in the mid-afternoon. Although there were some periods of significant difference between the photophobic rates of the three lights, the photophobic rates were generally low and stable for most of the time (Figure 3 and Figure S1).

Analysis of the significance of differences in photophilic and photophobic rates of *V. analis* at different times of the day showed that under ultraviolet light (360–365 nm), there were significant differences in photophilic and photophobic rates at every time of the day except the afternoon. Under ultraviolet light (380–385 nm), orange-red light, and red light, there were significant differences in photophilic and photophobic rates at each time of the day except in the morning and afternoon. Under violet, blue-violet, dark blue, blue, and greenish light, photophilic and photophobic rates were significantly different for all six time periods. Under bright green, yellow, and composite light, there was a significant difference in all periods except morning. Under green and green-yellow light, significant differences were found only in the morning, mid-day, and late afternoon. Overall, significant differences in photophilic and photophobic rates were found mainly in the morning and evening (Figure S1 and Table S4).

### 3.4. Phototactic Behavior of Vespa analis under Different Wavelengths of Light

The total activity rate of *Vespa analis* varies greatly throughout the day. Not all phototactic behavior data from each period can adequately reflect the phototactic behavior characteristics of *V. analis*. Therefore, we selected three of the six time periods with the highest total activity rate of *V. analis* to investigate the phototactic behavior of different wavelengths of light (Figure S1). It was found that *V. analis* exhibited phototactic responses to all 14 types of light. However, there were significant differences in the photophilic rate between wavelengths, suggesting that *V. analis* had varying degrees of sensitivity and preference for different wavelengths of light. In this study, it was found that *V. analis* had the lowest photophilic rate of 14.2% when exposed to ultraviolet light. White light had a photophilic rate of less than 30%, which was significantly higher than that of ultraviolet light. Violet and yellow light showed a further increase in preference compared to the previous light. Blue, green, and red lights were the most preferred lights by *V. analis*, with an average photophilic rate of about 40% (Table 2).

In conclusion, the comparison of photophilic, photophobic, and the total activity rate of *V. analis* in different wavelengths of light revealed significant differences in 14 light types (Table 3). The photophilic behavior of *V. analis* was much stronger than its photophobic behavior. When comparing the phototactic rate and the total activity rate of *V. analis*, a significant difference was found only under 380–385 nm UV light (Table S5). The total activity rate of *V. analis* was mainly dependent on the photophilic rate. The photophobic behavior was weak in most light conditions. The comparison between the photophobic rate and the total activity rate of *V. analis* revealed significant differences across all 14 lights (Table S6).

### 3.5. Analyses of Optic Protein Genes in Vespa basalis

#### 3.5.1. Physico-Chemical Characterization of Opsin Genes in *Vespa basalis*

The opsin genes Vb-BL and Vb-UV were obtained from *Vespa basalis* and submitted to the GenBank database. The Vb-BL and Vb-UV fragments contain 1775 and 1641 bases, with an open reading frame of 1137 and 1122 bases that encode a polypeptide comprising 378 and 373 amino acids. The predicted isoelectric point of the opsin genes was 8.40 and 8.02, and the Molecular weights were 42.98 and 41.45 kDa, respectively (Table S7). The functional sites of Vb-BL and Vb-UV all contained an N-glycosylation site, a protein kinase C phosphorylation site, a casein kinase II phosphorylation site, an N-myristoylation site, and a visual pigments (opsins) retinal binding site; however, Vb-UV also contains G-protein coupled receptors family 1 signature (Table S8).

#### 3.5.2. Predictive Analysis of the Structure of the Opsin Genes in *Vespa basalis*

**Figure 4:**
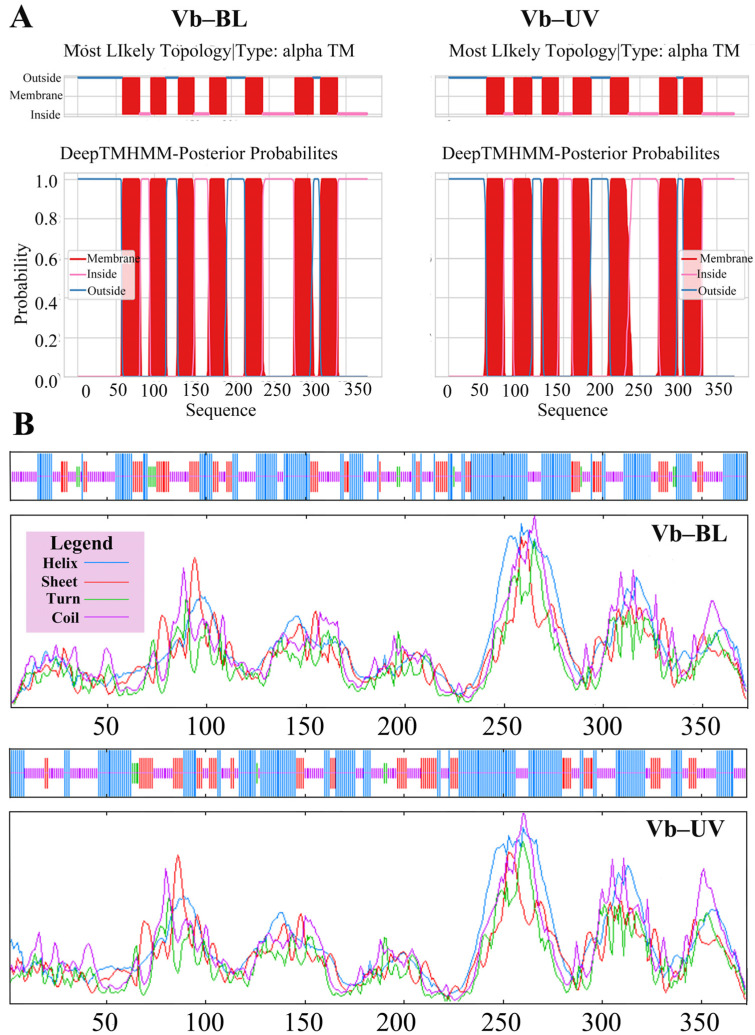
Analysis of opsin genes in *V. basalis*: (**A**) predicted transmembrane topology models of Vb-BL and Vb-UV and (**B**) the predicted secondary structure of Vb-BL and Vb-UV.

The transmembrane topologies of Vb-BL and Vb-UV were predicted. Vb-BL’s amino acid sequence was found to have seven transmembrane topologies located at positions 58–81, 95–115, 131–152, 172–194, 219–241, 283–307, and 317–339. Four topologies are from the outside to the inside, and three are from the inside to the outside (Figure 4B). The amino acid sequence of Vb-UV has seven transmembrane topologies located at positions 49–72, 85–109, 122–143, 162–186, 211–235, 275–275, and 275–298, respectively. Four of these transmembrane topologies are oriented from the outside in, while the remaining three are oriented from the inside out (Figure 4A).

The Secondary structure predictions for Vb-BL and Vb-UV indicate that they consist of approximately 41.80% and 44.24% alpha helix (Hh), 17.46% and 17.69% extended strand (Ee), 3.17% and 1.88% beta-turn (Tt), and 37.57% and 36.19% random coil (Cc) respectively (Figure 4B).

**Figure 5:**
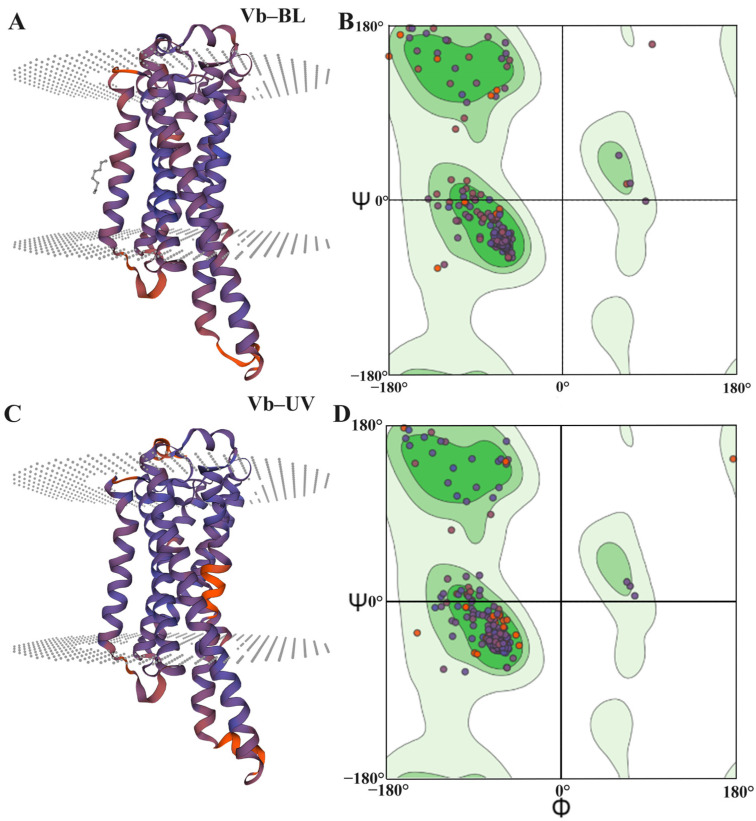
The 3D structures and Ramachandran plots of Vb−BL and Vb−UV in *V. basalis*: (**A**) the 3D structure of Vb−BL; (**B**) Ramachandran plot of Vb−BL; (**C**) the 3D structure of Vb−UV; and (**D**) Ramachandran plot of Vb−UV.

The 3D structure models of Vb-BL and Vb-UV of *V. basalis* were constructed using (Template) 6i9k.1.A (2.1 Å) as a template with GMQE values of 0.63 and 0.67, QMEAN values of −4.64 and −4.51, and sequence identities of 33.33% and 40.82% (Figure 5). The possibility of the modeled 3D structural models of Vb-BL and Vb-UV was assessed by Ramachandran plots. This study indicates that the stereo conformations and dihedral angles of the 3D structural models of Vb-BL and Vb-UV meet the requirements of the two dihedral angles Ψ and Ω distributions (Figure 5), suggesting that the constructed 3D structures are reliable.

**Figure 6:**
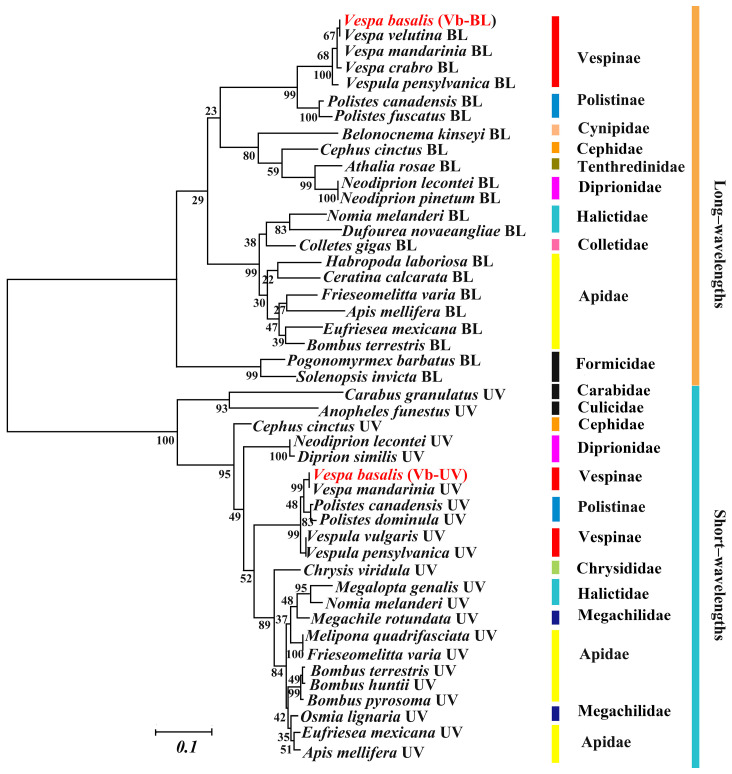
A maximum-likelihood (ML) tree of the opsin genes based on amino acid sequences. The other opsins represent the Hymenoptera species. Values above the nodes represent bootstrap values. Among them are long wavelengths (croci) and short wavelengths (blue). The red color indicates that these are newly sequenced genes.

The cloned *V. basalis* has two visual proteins, one short-wavelength and one long-wavelength. The phylogenetic tree for opsins from two groups, each with 22 insect species, showed that opsins were clustered with Vb−BL and Vb−UV opsins, respectively, and species within the same taxa (genus, family, and order) were also well clustered. The opsins of *V. basalis* were highly homologous to other vespids and were most closely related to its congeners (viz. *V. velutina*, *V. crabro*, and *V. mandarinia*) (Table S9). This suggests that opsin is conserved during evolution. Significantly, within the family Vespidae, the species of subfamily Vespinae (including the genera *Vespa* and *Vespula*) separated with the subfamily Polistinae (including the genus *Polistes*) clearly in long-wavelength opsins while mixed in short-wavelength opsins. Thinking of a few poor bootstrap value numbers in the tree, the phylogenetic relationships of these proteins were only to some degree consistent with traditional classification, indicating the limited reliability of the clustering relationships in the phylogenetic tree (Figure 6).

## 4. Discussion

The photophobic rate of *Vespula germanica* and *Vespa analis* was consistently lower than the photophilic rate, suggesting a weak aversion to light. This phenomenon may be attributed to the circadian rhythms of *Vespula* species. While not all *Vespula* species have identical activity patterns, they generally initiate their activities early in the morning, peak in and out of the hive around 11:30 a.m., and experience a significant decrease in activity in the evening (between 5:00 p.m. and 8:00 p.m.) [34,46]. The circadian rhythms of *V. mandarinia*, *V. basalis*, and *V. velutina* in Shaanxi Province, China, are generally similar, with activity commencing around 5:00 a.m. in the summer, increasing from 7:00 a.m. and then dramatically decreasing after returning to the nest at around 6:00 p.m. [47]. In September, *V. orientalis* in Israel exhibited increased activity in the early morning, with frequent movements in and out of the nest from 11:00 to 19:00, followed by a rapid decline in activity [48]. Overall, the influence of different periods on the phototactic behavior of *V. germanica* and *V. analis* aligns with their respective circadian rhythms.

Currently, a comprehensive explanation for insect phototactic behavior remains elusive. Although various hypotheses have been proposed [16,21,49], they are limited to specific types or classes of insect phototactic behavior. They do not fully elucidate the underlying mechanisms of dynamic phototactic behavior in insects. The study of insect phototactic behavior has progressed with advancements in biotechnology. Through scanning electron microscopy of the compound eye, retinal potentials, and other studies, many species’s light-sensitive peaks and the basic structure of the insect compound eye have been elucidated [50,51]. Molecular biology-based investigations of visual genes in insects hold promise for unraveling insect phototactic behavior [52,53].

The phototactic responses of *V. germanica* and *V. analis* varied across 14 different light wavelengths, indicating their sensitivity variations. For instance, *V. germanica* exhibited the highest sensitivity to violet light, while *V. analis* was most sensitive to red-orange light. However, their maximum phototactic rate reached only about 50%. Notably, they displayed low sensitivity to ultraviolet light, and their photophobic behavior was insignificant. Research has shown that Hymenoptera insects possess a trichromatic visual system, with spectral sensitivity peaks at 340 nm, 430 nm, and 535 nm [8]. This indicated the presence of UV-sensitive, blue-light-sensitive, and long-wave-sensitive retinoblast genes in insect eyes. For example, *Aphidius gifuensis* is sensitive to UV light at 330–340 nm, green light at 490 nm, and blue light at 530 nm [54]. *Apis mellifera* has three phototactically sensitive bands at 344 nm, 436 nm, and 556 nm, corresponding to their trichromatic visual system. However, unlikely honeybees and Vespinae possess only two retinoblast genes [9,55].

In general, insect retinoid genes are classified into three categories: ultraviolet-sensitive opsin, blue-sensitive opsin, and long-wave-sensitive opsin [56]. However, the cloning procedure of *V. basalis* yielded only two opsin genes, blue-sensitive opsin (Vb-BL) and ultraviolet-sensitive opsin (Vb-UV) [9,51], which represents a departure from the general situation [57,58]. Under the trichromatic color vision principle, insects with only two photoreceptor genes, such as hornets, would be expected to perceive a color-poor world, which is often referred to as color blindness. However, the absence of a gene for long-wavelength photoreceptor protein does not imply that wasps cannot perceive long-wavelength light. Conversely, the presence of a gene for a specific photoreceptor protein does not necessarily indicate that wasps exhibit a preference for that wavelength. Our study provides empirical evidence that supports this view; for example, the phototropic behavior of *V. germanica* and *V. analis* demonstrated that both species exhibited a phototropic peak in long-wavelength light, with the lowest phototropic rate observed for ultraviolet light. This suggests the potential for a more intricate regulatory mechanism underlying the influence of the visual protein gene on the phototactic behavior of wasps.

Insects have evolved visual systems that are adapted to their specific habitats. Many insects, such as *Helicoverpa armigera*, *Ostrinia furnacalis*, and *Chilo suppressalis*, exhibit clear positive phototactic behavior towards light [1,59] and some negative phototaxis (avoidance of light) [18]. As a result, light traps have been widely utilized in integrated pest management (IPM) for predicting and controlling various insect pests [60]. Diurnal insects have developed intricate visual systems that utilize ambient light for navigation and color vision [25,61]. Vision plays a crucial role in visual perception, circadian rhythm regulation, and pupil response in insects [62]. A detailed analysis of opsins, light-sensitive proteins, in insects is of great importance for understanding the mechanisms behind phototactic behavior and for enhancing the efficiency of light traps in attracting target insect pests.

## 5. Conclusions

This study provides valuable insights into the phototactic behavior of Vespinae species and their associated opsin genes. The findings indicate that the photophilic rates of *Vespula germanica* and *Vespa analis* are influenced by their circadian rhythms, with lower photophobic rates suggesting a weaker aversion to light. The observed variations in phototactic responses across different light wavelengths suggest species-specific sensitivities. *V. germanica* exhibited high sensitivity to violet light, while *V. analis* showed greater sensitivity to red-orange light. However, both species displayed low sensitivity to ultraviolet light. Therefore, this study follows a previous study highlighting the presence of a trichromatic visual system in Hymenoptera insects, with spectral sensitivity peaks at ultraviolet, blue, and long-wave wavelengths. These findings align with previous research on other insect species and reinforce the importance of retinoblast genes in mediating phototactic behavior. The research also emphasizes the potential of molecular biology-based investigations to further unravel the mechanisms underlying insect phototaxis. Insects exhibit diverse phototactic behaviors that are influenced by their ecological niche and behaviors. The compound eyes of insects play a vital role in their light responsiveness. Light traps utilizing positive phototaxis have proven effective in integrated pest management strategies. Understanding the visual systems and opsins in insects can enhance the efficiency of such traps in attracting target insect pests.

## Figures and Tables

**Table 1:** Primers for cloning opsin genes.

Primer Name	Primer Sequence 5′-3′
Vb-BL-F1	CATTCGATCTGTTTGATATAAAAGAGC
Vb-BL-R1	CTTTAGCGATTCTAACTTCGACACT
Vb-BL-F2	GGGATACCTAACTACGTGCAGTTTC
Vb-BL-R2	TCCATTTGATGCTTGCTTTATTC
Vb-UV-F1	ATCTTTTAAAAAAGAACCGATTTGTTG
Vb-UV-R1	GGTATCGGTTAAATAATCAAAAGAACAG
Vb-UV-F2	ATGGAAAATTATCACGTGGTCAAGT
Vb-UV-R2	TTATATGCCAGGAATTTACTTTATTGG

**Table 2:** Photophilic, photophobic, and total activity rate of *Vespula germanica* and *Vespa analis* under different wavelengths of light.

Species	*V. germanica* and *V. analis*	*V. germanica*	*V. analis*
Type	*p*-Value	F (DFn, DFd) = F(13, 154)	F (DFn, DFd) = F(13, 154)
Photophilic rate	<0.0001	13.12	18.11
Photophobic rate	<0.0001	5.746	6.891
Total activity rate	<0.0001	12.41	13.46

Abbreviations: *Vl*. = *Vespula*; *V.* = *Vespa*.

**Table 3:** Comparison with phototactic behavior of *Vespula germanica* and *Vespa analis* under different wavelengths of light.

Type	*Vespula germanica*			*Vespa analis*		
	Photophilic /Photophobic Rate	Photophobic /Total Activity Rate	Photophobic /Total Activity Rate	Photophilic /Photophobic Rate	Photophobic /Total Activity Rate	Photophobic /Total Activity Rate
360–365 nm	****	ns	****	****	ns	****
380–385 nm	****	ns	****	*	*	****
400–410 nm	****	ns	****	****	ns	****
420–430 nm	****	ns	****	****	ns	****
440–445 nm	****	ns	****	****	ns	****
460–475 nm	****	ns	****	****	ns	****
490–505 nm	****	ns	****	****	ns	****
515–525 nm	****	ns	****	****	ns	****
530–545 nm	****	*	****	****	ns	****
570–590 nm	****	ns	****	****	ns	****
600–610 nm	****	ns	****	****	ns	****
625–635 nm	****	ns	****	****	ns	****
625–635 nm	****	ns	****	****	ns	****
400–840 nm	****	ns	****	****	ns	****

The asterisk (*) indicates the significance level of the test result. The symbol “ns” represents non-marked differences, whereas a single * and **** represent statistically marked differences at 5% and 0.01%, respectively.

## Data Availability

The data are contained in this article.

## Supplementary Materials

The following supporting information can be downloaded at https://www.mdpi.com/article/10.3390/ani14111543/s1. The data presented in this study are contained within the article or Supplementary Materials. Figure S1: Photophilic rate, photophobic rate, and total activity rate of *Vespa analis* at different time intervals under monochromatic light. Table S1: Comparison of photophilic rate differences in *Vespula germanica* and *V. analis* at different time intervals under monochromatic light. Table S2: Comparison of photophilic rate differences in *V. germanica* and *V. analis* at different time intervals under monochromatic light. Table S3: Comparison of differences in total activity rate in *V. germanica* and *V. analis* at different time intervals under monochromatic light. Table S4: Comparison of differences in photophilic and photophobic rates in *V. germanica* and *V. analis* at different time intervals. Table S5: Comparison of differences in photophilic rate and total activity rate in *V. germanica* and *V. analis* at different time intervals. Table S6: Comparison of differences in photophobic rate and total activity rate in *V. germanica* and *V. analis* at different time intervals. Table S7: Sequence features of two opsin genes and their encoded amino acids with *V. basalis*. Table S8: The functional site analysis of Vb-BL and Vb-UV with *V. basalis*. Table S9: The comparative alignment of the amino acid sequence of Vb-BL with other homologous species.

## References

[1] Kim K.N., Huang Q.Y., Lei C.L. (2019). Advances in insect phototaxis and application to pest management: A review. Pest. Manag. Sci..

[2] Neckameyer W.S., Bhatt P. (2016). Protocols to Study Behavior in *Drosophila*. Methods Mol. Biol..

[3] Pan H., Liang G., Lu Y. (2021). Response of Different Insect Groups to Various Wavelengths of Light under Field Conditions. Insects.

[4] Hay D., Crossley S. (1977). The design of mazes to study *Drosophila* behavior. Behav. Genet..

[5] Paris T.M., Allan S.A., Udell B.J., Stansly P.A. (2017). Wavelength and polarization affect phototaxis of the Asian Citrus Psyllid. Insects.

[6] Yao M.C., Lee C.Y., Chiu H.W., Feng W.B., Yang E.C., Lu K.H. (2022). Efficiency of a Novel Light-Emitting Diode (LED) Trap for Trapping *Rhyzopertha dominica* (Coleoptera: Bostrichidae) in Paddy Rice Storehouses. J. Econ. Entomol..

[7] Muri R.B., Jones G.J. (1983). Microspectrophotometry of single rhabdoms in the retina of the honeybee drone (*Apis mellifera* male). J. Gen. Physiol..

[8] Peitsch D., Fietz A., Hertel H. (1992). The spectral input systems of hymenopteran insects and their receptor-based colour vision. J. Comp. Physiol..

[9] Menzel R., Blakers M. (1976). Colour receptors in the bee eye-morphology and spectral sensitivity. J. Comp. Physiol..

[10] Hecht S., Wald G. (1934). The visual acuity and intensity discrimination of *Drosophila*. J. Comp. Physiol..

[11] Chen Z., Kuang P.R., Zhou J.X. (2012). Phototactic behavior in *Aphidius gifuensis* (Hymenoptera: Braconidae). Biocontrol Sci. Technol..

[12] Kim J.G., Lee E.H., Seo Y.M., Kim N.Y. (2011). Cyclic behavior of *Lycorma delicatula* (Insecta: Hemiptera: Fulgoridae) on host plants. J. Insect Behav..

[13] Menzel R., Backhaus W. (1992). Color Vision in Insects.

[14] Nouvian M., Galizia C.G. (2020). Complexity and plasticity in honey bee phototactic behavior. Sci. Rep..

[15] Campagna C., Fernandez T.A. (2007). comparative analysis of the vision and mission statements of international environmental organisations. Environ. Values.

[16] Park Y.G., Lee Y.S., Sarker S., Ham E.H., Lim U.T. (2023). Attractiveness of four wavelengths of LED light: UV (385 nm), violet (405 nm), blue (450 nm), and red (660 nm) for seven species of natural enemies. Biol. Control.

[17] Cheng W.J., Zheng X.L., Wang P., Zhou L.L., Si S.Y., Wang X.P. (2016). Male-Biased Capture in Light Traps in *Spodoptera exigua* (Lepidoptera: Noctuidae): Results from the Studies of Reproductive Activities. J. Insect Behav..

[18] Yang E.C., Lee D.W., Wu W.Y. (2003). Action spectra of phototactic responses of the flea beetle, *Phyllotreta striolata*. Physiol. Entomol..

[19] Sun G., Liu S., Luo H., Feng Z., Yang B., Luo J., Tang J., Yao Q., Xu J. (2022). Intelligent Monitoring System of Migratory Pests Based on Searchlight Trap and Machine Vision. Front. Plant Sci..

[20] Robinson H.S. (1952). On the behaviour of night-flying insects in the neighbourhood of a bright source of light. Entomol. Gen..

[21] Callahan P.S. (1965). Intermediate and far infrared sensing of nocturnal insects. Ann. Entomol. Soc. Am..

[22] Michael D.A. (1980). Introduction to Insect Behavior.

[23] Sang W., Huang Q., Wang X., Guo S.H., Lei C.L. (2019). Development, achievement, and prospect of insect phototaxis and light trapping techniques in China. J. Appl. Entomol..

[24] Gebhardt F., Desplan C. (2017). Retinal perception and ecological significance of color vision in insects. Curr. Opin. Insect Sci..

[25] Arikawa K., Stavenga D.G. (2014). Insect photopigments: Photoreceptor spectral sensitivities and visual adaptations. Evolution of Visual and Non-Visual Pigments.

[26] Terakita A. (2005). The opsins. Genome Biol..

[27] Yuan F., Bernard G.D., Le J. (2010). Contrasting modes of evolution of the visual pigments in *Heliconius* butterflies. Mol. Biol. Evol..

[28] Henze M.J., Oakley T.H. (2015). The dynamic evolutionary history of pancrustacean eyes and opsins. Integr. Comp. Biol..

[29] Oba Y., Kainuma T. (2009). Diel changes in the expression of long wavelength-sensitive and ultraviolet-sensitive opsin genes in the Japanese firefly, *Luciola cruciata*. Gene.

[30] Briscoe A.D., Bybee S.M., Bernard G.D. (2010). Positive selection of a duplicated UV-sensitive visual pigment coincides with wing pigment evolution in *Heliconius* butterflies. Proc. Natl. Acad. Sci. USA.

[31] Dyer A.G., Chittka L. (2004). Bumblebee search time without ultraviolet light. J. Exp. Biol..

[32] Froy O., Gotter A.L., Casselman A.L. (2003). Illuminating the circadian clock in monarch butterfly migration. Science.

[33] Li C.F., Tian F.J., Lin T., Wang Z.B., Liu J.L., Zeng X.N. (2000). The expression and function of opsin genes related to the phototactic behavior of Asian citrus psyllid. Pest Manag. Sci..

[34] Tan J.L., van Achterberg C., Chen X.X. (2015). Potentially Lethal Social Wasps, Fauna of the Chinese Vespinae (Hymenoptera: Vespidae).

[35] Kogan M. (1998). Integrated pest management: Historical perspectives and contemporary developments. Annu. Rev. Entomol..

[36] Wilson R., Wakefield A., Roberts N., Jones G. (2021). Artificial light and biting flies: The parallel development of attractive light traps and unattractive domestic lights. Parasites Vectors.

[37] Huang M., Meng J.Y., Zhou L.C., Zhang Y., Yu C. (2023). Expression and function of opsin genes associated with phototaxis in Zeugodacus cucurbitae Coquillett (Diptera: Tephritidae). Pest Manag. Sci..

[38] Jiang X., Hai X., Bi Y., Zhao F., Wang Z., Lyu F. (2023). Research on Photoinduction-Based Technology for Trapping Asian Longhorned Beetle (*Anoplophora glabripennis*) (Motschulsky, 1853) (Coleoptera: Cerambycidae). Insects.

[39] Wu Y.Q., Jiang Y.L., Zhou G.T., Zhang G.P., Miao J., Gong Z.J., Duan Y., Li T. (2023). A review of studies of insect phototaxis. J. Environ. Entomol..

[40] Rode J.B., Ringel M.M. (2019). Statistical Software Output in the Classroom: A Comparison of R and SPSS. Teach. Psychol..

[41] Gasteiger E., Gattiker A., Hoogland C., Ivanyi I., Appel R.D., Barioch A. (2005). ExPASy: The proteomics server for in-depth protein knowledge and analysis. Nucleic Acids Res..

[42] Hallgren J., Tsirigos K.D., Pedersen M.D. (2022). DeepTMHMM predicts alpha and beta transmembrane proteins using deep neural networks. bioRxiv.

[43] Waterhouse A., Bertoni M., Bienert S. (2018). SWISS-MODEL: Homology modeling of protein structures and complexes. Nucleic Acids Res..

[44] Notredame C., Higgins D.G., Heringa J. (2000). T–Coffee: A novel method for fast and accurate multiple sequence alignment. J. Mol. Biol..

[45] Tamura K., Stecher G., Kumar S. (2021). MEGA11: Molecular evolutionary genetics analysis version 11. Mol. Biol. Evol..

[46] Lester P.J., Beggs J.R. (2019). Invasion Success and Management Strategies for Social *Vespula* Wasps. Annu. Rev. Entomol..

[47] Dyson C.J., Crossley H.G., Ray C.H., Goodisman M.A.D. (2022). Social structure of perennial Vespula squamosa wasp colonies. Ecol. Evol..

[48] Ishay J. (1967). Contributions to the bionomics of the Oriental hornet *Vespa orientalis* F. Isr. J. Entomol..

[49] Wolf E., Zerrahn-Wolf G. (1935). The effect of light intensity, area, and flicker frequency on the visual reactions of the honey bee. J. Gen. Physiol..

[50] Makarova A., Polilov A., Fischer S. (2015). Comparative morphological analysis of compound eye miniaturization in minute hymenoptera. Arthropod Struct. Dev..

[51] Dai B., Zhang L., Zhao C., Bachman H., Becker R., Mai J., Jiao Z., Li W., Zheng L., Wan X. (2021). Biomimetic apposition compound eye fabricated using microfluidic-assisted 3D printing. Nat. Commun..

[52] Wang Y., Fang G., Xu P., Gao B., Liu X., Qi X., Zhang G., Cao S., Li Z., Ren X. (2022). Behavioral and genomic divergence between a generalist and a specialist fly. Cell Rep..

[53] Mishra M., Knust E. (2013). Analysis of the *Drosophila* compound eye with light and electron microscopy. Methods Mol. Biol..

[54] Song Y., Liu C., Cai P., Chen W., Guo Y., Lin J., Zhang S. (2021). Host-Seeking Behavior of *Aphidius gifuensis* (Hymenoptera: Braconidae) Modulated by Chemical Cues Within a Tritrophic Context. J. Insect Sci..

[55] Menzel R., Backhaus W. (2022). Colour vision in nocturnal insects. Philos. Trans. R. Soc. B Biol. Sci..

[56] Briscoe A.D., Chittka L. (2001). The evolution of color vision in insects. Annu. Rev. Entomol..

[57] Briscoe A.D. (2000). Six opsins from the butterfly *Papilio glaucus*: Molecular phylogenetic evidence for paralogous origins of red-sensitive visual pigments in insects. J. Mol. Evol..

[58] Ogawa Y., Kinoshita M., Stavenga D.G. (2013). Sex-specific retinal pigmentation results in sexually dimorphic long-wavelength-sensitive photoreceptors in the eastern pale clouded yellow butterfly, *Colias erate*. J. Exp. Biol..

[59] Su L., Yang C.L., Meng J.Y., Zhou L., Zhang C.Y. (2021). Comparative transcriptome and metabolome analysis of *Ostrinia furnacalis* female adults under UV-A exposure. Sci. Rep..

[60] Meng J.Y., Zhang C.Y., Zhu F., Wang X.P., Lei C.L. (2009). Ultraviolet light induced oxidative stress: Effects on antioxidant response of *Helicoverpa armigera* adults. J. Insect Physiol..

[61] Sauman I., Briscoe A.D., Zhu H., Shi D., Froy O., Stalleicken J., Yuan Q., Casselman A., Reppert S.M. (2005). Connecting the navigational clock to sun compass input in monarch butterfly brain. Neuron.

[62] Koyanagi M., Terakita A. (2014). Diversity of animal opsin-based pigments and their optogenetic potential. BBA Bioenerg..

