# Supplementary material for: Investigating the Influence of Varied Light-Emitting Diode (LED) Wavelengths on Phototactic Behavior and Opsin Genes in Vespinae
Source: Animals (Basel). 2024 May 23;14(11):1543. doi: 10.3390/ani14111543 (PMC11171232; doi:10.3390/ani14111543)
Supplement: Supplementary file 1 [file animals-14-01543-s001.zip › animals-2983779-supplementary.pdf]

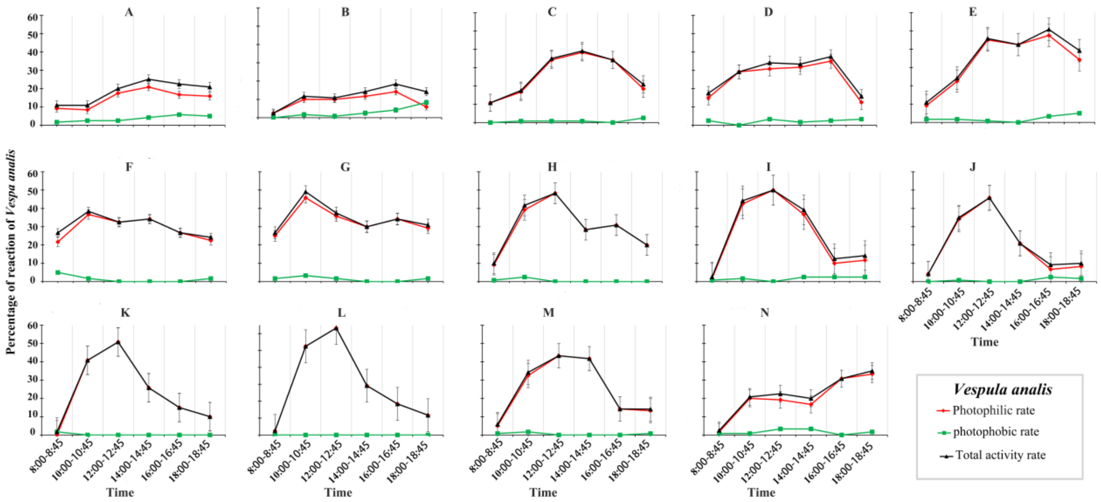

Figure S1. Photophilic rate, photophobic rate, and Total activity rate of *Vespa analis* at different time intervals under monochromatic light. (A) 360 nm-365 nm; (B) 380 nm-385 nm; (C) 400 nm-410 nm; (D) 420 nm-430 nm; (E) 440 nm-445 nm; (F) 460 nm-475 nm; (G) 490 nm-505 nm; (H) 515 nm-525 nm; (I) 530 nm-545 nm; (J) 550 nm-565 nm; (K) 570 nm-590 nm; (L) 600 nm-610 nm; (M) 625 nm-635 nm; (N) 400 nm-840 nm.

Table S1. Comparison of photophilic rate differences of *Vespula germanica* and *Vespa analis* different time intervals under monochromatic light.

Type	F (DFn, DFd)	P value	Significance level
360 -365 nm	F (5, 18) = 6.630[a]/ 5.267[b]	0.0012[a]/0.0037[b]	**[ab]
380 -385 nm	F (5, 18) = 4.597[a]/ 8.639[b]	0.0071[a]/0.0003[b]	**[a]/***[b]
400 -410 nm	F (5, 18) = 6.286[a]/30.16[b]	0.0015[a]/<0.0001[b]	**[a]/****[b]
420 -430 nm	F (5, 18) = 16.44[a]/38.11[b]	<0.0001[ab]	****[ab]
440 -445 nm	F (5, 18) = 9.691[a]/37.50[b]	0.0001[ab]	***[ab]
460 -475 nm	F (5, 18) = 29.88[a]/4.082[b]	<0.0001[a]/0.0119[b]	****[a]/*[b]
490 -505 nm	F (5, 18) = 6.182[a]/6.488[b]	<0.0017[a]/0.0013[b]	**[a]/**[b]
515 -525 nm	F (5, 18) = 16.78[a]/ 19.22[b]	<0.0001[ab]	****[ab]
530 -545 nm	F (5, 18) = 13.22[a]/35.82[b]	<0.0001[ab]	****[ab]
550 -565 nm	F (5, 18) = 38.62[a]/35.57[b]	<0.0001[ab]	****[ab]
570 -590 nm	F (5, 18) = 32.09[a]/88.50[b]	<0.0001[ab]	****[ab]
600 -610 nm	F (5, 18) = 29.79[a]/55.69[b]	<0.0001[ab]	****[ab]
625 -635 nm	F (5, 18) = 9.047[a]/27.90[b]	<0.0002[a]/<0.0001[b]	***[a]/****[b]
400 -840 nm	F (5, 18) = 16.27[a]/50.32[b]	<0.0001[ab]	****[ab]

[a] *Vespula germanica*; [b] *Vespa analis*. The asterisk (*) indicates the significance level of the test result. The symbol "ns" is used to represent non-marked differences, whereas a single *, **, ***, and **** is used to represent statistically marked differences at 5%, 1%, 0.1%, and 0.01% levels, respectively.

Table S2. Comparison of photophobic rate differences of *Vespula germanica* and *Vespa analis* at different time intervals under monochromatic light.

Type	F (DFn, DFd)	P value	Significance level
360 -365 nm	F (5, 18) = 1.800[a]/1.580[b]	0.1637[a]/0.2160[b]	ns[ab]
380 -385 nm	F (5, 18) = 3.079[a]/2.647[b]	0.0351[a]/0.0580[b]	*[a]/ns[b]
400 -410 nm	F (5, 18) = 0.6537[a]/1.085[b]	0.6626[a]/0.4017[b]	ns[ab]
420 -430 nm	F (5, 18) = 1.530[a]/1.551[b]	0.2301[a]/0.2242[b]	ns[ab]
440 -445 nm	F (5, 18) = 1.084[a]/2.176[b]	0.4024[a]/0.1025[b]	ns[ab]
460 -475 nm	F (5, 18) = 1.761[a]/3.521[b]	0.1720[a]/0.0215[b]	ns[a]/*[b]
490 -505 nm	F (5, 18) = 1.200[a]/1.446[b]	0.3485[a]/0.2557[b]	ns[ab]
515 -525 nm	F (5, 18) = 0.4757[a]/4.400[b]	0.7896[a]/0.0086[b]	ns[a]/**[b]
530 -545 nm	F (5, 18) = 4.749[a]/1.194[b]	0.0061[a]/0.3512[b]	**[a]/ns[b]
550 -565 nm	F (5, 18) = 0.8000[a]/1.597[b]	0.5640[a]/0.2114[b]	ns[ab]
570 -590 nm	F (5, 18) = 1.391[a]/1.000[b]	0.2742[a]/0.4457[b]	ns[ab]
600 -610 nm	F (5, 18) = 2.407[a]/1.000[b]	0.0773[a]/0.4457[b]	ns[ab]
625 -635 nm	F (5, 18) = 0.6440[a]/1.200[b]	0.6694[a]/0.3485[b]	ns[ab]
400 -840 nm	F (5, 18) = 3.262[a]/2.786[b]	0.0286[a]/0.0492[b]	*[a]/ns[b]

[a] *Vespula germanica*; [b] *Vespa analis*. The asterisk (*) indicates the significance level of the test result. The symbol "ns" is used to represent non-marked differences, whereas a single *, **, ***, and **** is used to represent statistically marked differences at 5%, 1%, 0.1%, and 0.01% levels, respectively.

Table S3. Comparison of differences in total activity rate of *Vespula germanica* and *Vespa analis* at different time intervals under monochromatic light.

Type	F (DFn, DFd)	P value	Significance level
360 -365 nm	F (5, 18) = 6.640[a]/5.180[b]	0.0011[a]/0.0041[b]	**[ab]
380 -385 nm	F (5, 18) = 5.416[a]/5.592[b]	0.0033[a]/0.0028[b]	**[ab]
400 -410 nm	F (5, 18) = 5.976[a]/ 25.69[b]	0.0020[a]/<0.0001[b]	**[a]/****[b]
420 -430 nm	F (5, 18) = 22.88[a]/24.87[b]	<0.0001[ab]	****[ab]
440 -445 nm	F (5, 18) = 9.822[a]/29.23[b]	0.0001[ab]	***[a]/****[b]
460 -475 nm	F (5, 18) = 26.56[a]/2.629[b]	<0.0001[a]/0.0593[b]	****[a]/ns[b]
490 -505 nm	F (5, 18) = 6.598[a]/8.405[b]	<0.0012[a]/0.0003[b]	**[a]/***[b]
515 -525 nm	F (5, 18) = 15.41[a]/18.62[b]	<0.0001[ab]	****[ab]
530 -545 nm	F (5, 18) = 8.938[a]/36.04[b]	<0.0002[a]/<0.0001[b]	***[a]/****[b]
550 -565 nm	F (5, 18) = 34.48[a]/29.22[b]	<0.0001[ab]	****[ab]
570 -590 nm	F (5, 18) = 28.04[a]/76.33[b]	<0.0001[ab]	****[ab]
600 -610 nm	F (5, 18) = 31.48[a]/55.69[b]	<0.0001[ab]	****[ab]
625 -635 nm	F (5, 18) = 7.860[a]/25.52[b]	0.0004[a]/<0.0001[b]	***[ab]
400 -840 nm	F (5, 18) = 8.496[a]/31.46[b]	0.0003[ab]	***[ab]

[a] *Vespula germanica*; [b] *Vespa analis*. The asterisk (*) indicates the significance level of the test result. The symbol "ns" is used to represent non-marked differences, whereas a single *, **, ***, and **** is used to represent statistically marked differences at 5%, 1%, 0.1%, and 0.01% levels, respectively.

Table S4. Comparison of differences in photophilic and photophobic rate of *Vespula germanica* and *Vespa analis* at different time intervals.

Type	A	B	C	D	E	F
360 -365 nm	ns[a]/**[b]	ns[a]/*[b]	****[a]/***[b]	**[ab]	ns[ab]	ns[a]/**[b]
380 -385 nm	ns[ab]	ns[a]/*[b]	**[ab]	ns[a]/*[b]	*[a]/**[b]	ns[a]/ns[b]
400 -410 nm	ns[a]/*[b]	*[a]/***[b]	****[ab]	****[ab]	***[a]/****[b]	**[a]/****[b]
420 -430 nm	ns[a]/***[b]	***[a]/****[b]	***[a]/****[b]	****[ab]	**[a]/****[b]	****[a]/*[b]
440 -445 nm	ns[a]/*[b]	*[a]/***[b]	*[a]/****[b]	***[a]/**[b]	****[a]/****[b]	***[a]/****[b]
460 -475 nm	ns[a]/*[b]	*[a]/***[b]	**[a]/****[b]	****[ab]	***[a]/****[b]	**[a]/****[b]
490 -505 nm	ns[a]/***[b]	ns[a]/****[b]	****[ab]	*[a]/***[b]	ns[a]/****[b]	*[a]/****[b]
515 -525 nm	ns[ab]	*[a]/****[b]	****[ab]	****[a]/***[b]	ns[a]/****[b]	***[a]/***[b]
530 -545 nm	*[a]/ns[b]	ns[a]/****[b]	***[a]/****[b]	**[a]/**[b]	ns[ab]	*[a]/ns[b]
550 -565 nm	ns[ab]	ns[a]/****[b]	****[ab]	****[a]/**[b]	****[a]/ns[b]	****[a]/ns[b]
570 -590 nm	ns[ab]	***[a]/****[b]	****[ab]	****[ab]	****[a]/***[b]	***[a]/***[b]
600 -610 nm	ns[ab]	ns[a]/****[b]	***[a]/****[b]	****[ab]	****[ab]	****[a]/ns[b]
625 -635 nm	ns[ab]	ns[a]/****[b]	****[ab]	***[a]/****[b]	****[a]/*[b]	ns[a]/****[b]
400 -840 nm	ns[ab]	****[a]/****[b]	****[ab]	****[a]/**[b]	**[a]/****[b]	ns[a]/****[b]

[a] *Vespula germanica*; [b] *Vespa analis*. The asterisk (*) indicates the significance level of the test result. The symbol "ns" is used to represent non-marked differences, whereas a single *, **, ***, and **** is used to represent statistically marked differences at 5%, 1%, 0.1%, and 0.01% levels, respectively; A: 8:00-8:45; B: 10:00-10:45; C: 12:00-12:45; D: 14:00-14:45; E: 16:00-16:45; F: 18:00-18:45.

Table S5. Comparison of differences in photophilic rate and total activity rate of *Vespula germanica* and *Vespa analis* at different time intervals.

Type	A	B	C	D	E	F
360 -365 nm	ns[ab]	ns[ab]	ns[ab]	ns[ab]	ns[ab]	ns[ab]
380 -385 nm	ns[ab]	ns[ab]	ns[ab]	ns[ab]	ns[ab]	ns[ab]
400 -410 nm	ns[ab]	ns[ab]	ns[ab]	ns[ab]	ns[ab]	ns[ab]
420 -430 nm	ns[ab]	ns[ab]	ns[ab]	ns[ab]	ns[ab]	ns[ab]
440 -445 nm	ns[a]/	ns[ab]	ns[ab]	ns[ab]	ns[ab]	ns[ab]
460 -475 nm	ns[a]/	ns[ab]	ns[ab]	ns[ab]	ns[ab]	ns[ab]
490 -505 nm	ns[a]/	ns[ab]	ns[ab]	ns[ab]	ns[ab]	ns[ab]
515 -525 nm	ns[a]/	ns[ab]	ns[ab]	ns[ab]	ns[ab]	ns[ab]
530 -545 nm	**[a]/ns	***[a]/ns[b]	ns[ab]	ns[ab]	ns[ab]	ns[ab]
550 -565 nm	ns[ab]	ns[ab]	ns[ab]	ns[ab]	ns[ab]	ns[ab]
570 -590 nm	ns[ab]	ns[ab]	ns[ab]	ns[ab]	ns[ab]	ns[ab]
600 -610 nm	ns[ab]	ns[ab]	ns[ab]	ns[ab]	ns[ab]	ns[ab]
625 -635 nm	ns[ab]	ns[ab]	ns[ab]	ns[ab]	ns[ab]	ns[ab]
400 -840 nm	ns[ab]	ns[ab]	ns[a]/*[b]	ns[ab]	ns[ab]	ns[ab]

[a] *Vespula germanica*; [b] *Vespa analis*. The asterisk (*) indicates the significance level of the test result. The symbol "ns" is used to represent non-marked differences, whereas a single *, **, ***, and **** is used to represent statistically marked differences at 5%, 1%, 0.1%, and 0.01% levels, respectively; A: 8:00-8:45; B: 10:00-10:45; C: 12:00-12:45; D: 14:00-14:45; E: 16:00-16:45; F: 18:00-18:45.

Table S6. Comparison of differences in photophobic rate and total activity rate of *Vespula germanica* and *Vespa analis* at different time intervals.

Type	A	B	C	D	E	F
360 nm-365 nm	ns[a]/***[b]	ns[a]/**[b]	****[a]/***[b]	**[a]/***[b]	ns[a]/*[b]	ns[a]/***[b]
380 nm-385 nm	ns[ab]	ns[a]/**[b]	**[ab]	*[a]/**[b]	*[a]/***[b]	ns[a]/ns[b]
400 nm-410 nm	ns[a]/*[b]	*[a]/***[b]	****[ab]	****[ab]	***[a]/****[b]	**[a]/****[b]
420 nm-430 nm	ns[a]/****[b]	***[a]/****[b]	****[ab]	****[ab]	**[a]/****[b]	****[a]/**[b]
440 nm-445 nm	ns[a]/*[b]	*[a]/***[b]	*[a]/***[b]	****[ab]	****[ab]	***[a]/****[b]
460 nm-475 nm	ns[a]/**[b]	**[a]/***[b]	**[a]/***[b]	****[ab]	***[a]/****[b]	**[a]/****[b]
490 nm-505 nm	ns[a]/****[b]	ns[a]/****[b]	****[ab]	*[a]/***[b]	ns[a]/****[b]	**[a]/****[b]
515 nm-525 nm	ns[ab]	**[a]/****[b]	****[ab]	****[a]/***[b]	*[a]/****[b]	***[ab]
530 nm-545 nm	ns[ab]	****[ab]	****[ab]	***[a]/**[b]	*[a]/ns[b]	**[a]/ns[b]
550 nm-565 nm	ns[ab]	ns[a]/****[b]	****[ab]	****[a]/**[b]	****[a]/ns[b]	****[a]/ns[b]
570 nm-590 nm	ns[ab]	***[a]/****[b]	****[ab]	****[ab]	****[a]/***[b]	***[a]/***[b]
600 nm-610 nm	ns[ab]	ns[a]/****[b]	***[a]/***[b]	****[ab]	****[ab]	****[a]/ns[b]
625 nm-635 nm	ns[ab]	ns[a]/****[b]	****[ab]	***[a]/****	****[a]/*[b]	ns[ab]
400 nm-840 nm	ns[ab]	****[ab]	****[ab]	****[a]/***	**[a]/****	ns[ab]

[a] *Vespula germanica*; [b] *Vespa analis*. The asterisk (*) indicates the significance level of the test result. The symbol "ns" is used to represent non-marked differences, whereas a single *, **, ***, and **** is used to represent statistically marked differences at 5%, 1%, 0.1%, and 0.01% levels, respectively; A: 8:00-8:45; B: 10:00-10:45; C: 12:00-12:45; D: 14:00-14:45; E: 16:00-16:45; F: 18:00-18:45.

Table S7. Sequence features of two opsin genes and their encoded amino acids with *Vespa basalis*.

Gene name	ORF(bp)	Protein length (aa)	Molecular weight (kDa)	Isoelectric points (IP)
Va-BL	1137	378	42.98	8.40
Va-UV	1122	373	41.45	8.04

Table S8. The functional sites analysis of Va-BL and Va-UV with *Vespa basalis.*

Functional site	Va-BL		Va-UV	
	Site	Sequence signature	Site	Sequence signature
N-glycosylation site	5 - 8	NYTF	3 - 6	NDSI
	167 - 170	NGSQ		
	365 - 368	NVSA		
Protein kinase C phosphorylation site	84 - 86	TSK	78 - 80	SLR
	246 - 248	SIR	256 - 258	SLR
	370 - 372	TEK		
Casein kinase II phosphorylation site	268 - 271	SNQD	360 - 363	TTTE
	329 - 332	SCID		
N-myristoylation site	136 - 141	GSISGI	116 - 121	GNLACQ
	140 - 145	GIGQAI	127 - 132	GSLSGI
	142 - 147	GQAISN	131 - 136	GIGAAV
	168 - 173	GSQAGL	133 - 138	GAAVTN
	172 - 177	GLIVAF		
	305 - 310	GAFGNS	297 - 302	GAFGNK
	318 - 323	GMLPAV		
G-protein coupled receptors family 1 signature	-	-	133 - 149	GAAVTNAA I AYDRYSTI
Visual pigments (opsins) retinal binding site	320 - 336	LPAVFAKSVSCIDP WIY	312 - 328	IPACFCKSV A CIDPYVY
	5 different patterns found		5 different patterns found	

Table S9. The comparative alignment of the amino acid sequence of Va-BL and Va-UV with other homologous species.

Va-BL			Va-UV		
Species	Number	Comparison rates (%)	Species	Number	Comparison rates (%)
Vespa velutina	XP_047359956	99.47	*Polistes dominula*	XP_015176771	97.05
Vespa mandarinia	XP_035721360	99.74	*Vespa mandarinia*	XP_035727087	100
Vespa crabro	XP_046827431	98.94	*Vespula vulgaris*	XP_050869610	98.39
Vespula pensylvanica	XP_043676321	97.62	*Vespula pensylvanica*	XP_043685726	98.12
Polistes canadensis	XP_014613625	83.39	*Polistes canadensis*	XP_014599192	97.32
Polistes fuscatus	XP_043505000	88.62	*Osmia lignaria*	XP_034183661	87.94
Neodiprion lecontei	XP_015516920	76.29	*Bombus pyrosoma*	XP_043591638	87.67
Neodiprion pinetum	XP_046469944	76.02	*Bombus huntii*	XP_050475658	87.13
Colletes gigas	XP_043260665	75.99	*Megachile rotundata*	XP_003699784	86.86
Athalia rosae	XP_012257660	75.82	*Frieseomelitta varia*	XP_043509454	86.33
Frieseomelitta varia	XP_043521129	75.20	*Melipona quadrifasciata*	KOX68034	86.06
Eufriesea mexicana	XP_017767215	74.67	*Nomia melanderi*	XP_031830873	84.45
Cephus cinctus	XP_015588548	74.65	*Cephus cinctus*	XP_015608081	87.40
Habropoda laboriosa	XP_017798773	74.14	*Eufriesea mexicana*	XP_017758468	85.79
Ceratina calcarata	XP_017884555	74.14	*Diprion similis*	XP_046737872	84.18
Bombus terrestris	XP_003400385	73.09	*Bombus terrestris*	XP_048265126	87.37
Belonocnema kinseyi	XP_033210608	73.02	*Chrysis viridula*	APY20525	84.99
Nomia melanderi	XP_031832874	72.82	*Neodiprion lecontei*	XP_015524256	84.72
Dufourea novaeangliae	KZC05676	71.24	*Megalopta genalis*	XP_033326020	84.45
Apis mellifera	NP_001011606	70.03	*Apis mellifera*	NP_001011605	84.99
Pogonomyrmex barbatus	XP_011638679	68.07	*Carabus granulatus*	APY20570	70.41
Solenopsis invicta	XP_011167017	66.49	*Anopheles funestus*	XP_049296566	66.49
